# New *p*-Substituted Salicylaldehyde Phenylhydrazone Derivatives: Synthesis, Characterization, and Antioxidant Activities

**DOI:** 10.3797/scipharm.1405-04

**Published:** 2014-07-01

**Authors:** Cigdem Yorur-Goreci, Nilay Altas-Kiymaz, Aysegul Peksel, Belkis Bilgin-Eran, Mustafa Sonmez

**Affiliations:** ^1^ Department of Organic Chemistry, Faculty of Arts and Sciences, Yildiz Technical University, 34210, Esenler/Istanbul, Turkey.; ^2^ Department of Biochemistry, Faculty of Arts and Sciences, Yildiz Technical University, 34210, Esenler/Istanbul, Turkey.

**Keywords:** Antioxidant activity, Metal chelating activity, Phenylhydrazones, Radical scavenging activity, Reducing power, Total antioxidant activity

## Abstract

A series of new p-nitrophenylhydrazone derivatives 3a–f were synthesized, characterized, and investigated for their antioxidant activities. These compounds have been synthesized by refluxing (p-nitrophenyl)hydrazine with 4-sub-stituted salicylaldehydes. The structures of the compounds were established by IR, ^1^H- and ^13^C-NMR, and MS data. The antioxidant activities (free radical-scavenging activity, reducing power, metal chelating activity, and total anti-oxidant activity) of the hydrazone compounds were evaluated. All of the compounds exhibited significant activities, while compound 3a, with the shortest chain, showed the highest antioxidant activity in all of the tests.

## Introduction

Hydrazones are important biological materials that have demonstrated diverse pharmacological activities such as antitumoral, antimicrobial, antimalarial, and anti-convulsant activities. The linkage –NH-N=CH- group of these compounds has a key role in various bioactive agents [1–5].

Free radicals are atoms or atom groups containing an unpaired electron. These radicals may cause a sequential series of reactions because of many active materials and can be triggered after a reaction step. The free radical species are reactive oxygen (ROS) and nitrogen (RNS) species, carbon-, and sulfur-centered radicals [6]. ROS are the superoxide, hydroxyl, and hydroperoxyl radicals as well as nitric oxide and other species such as hydrogen peroxide, peroxynitrite, singlet oxygen, and hypochlorous acid. The RNS species are also formed from the reaction of nitric oxide with singlet oxygen [7]. Antioxidants are important molecules which can react with the free radicals and block their sequential reactions without causing significant damage. The correlation of the production-neutralization of antioxidants with the reactive oxygen species is very sensitive. So, if the correlation causes too much production of ROS radicals, the cells suffer from the results of oxidative stress. Since oxidative stress is a key part of many diseases, antioxidant studies are done extensively in the pharmaceutical area [8, 9].

It is known from previous studies that phenylhydrazones of some ketones exhibit significant activities such as antitubercular and antiviral. Moreover, the salicylaldehyde benzoylhydrazone compounds inhibit DNA synthesis in cells of radiation-sensitive tissues [10]. Studies on free radical processes occurring in a living body have shown that phenyl-hydrazone compounds have an important pharmacological effect. The antioxidant activities of phenylhydrazone compounds have been studied over the past two decades [11, 12].

In this work, we studied the different antioxidant activities of phenylhydrazones and the correlation of their reactivity in radical reactions with their compositions and molecular structures.

With the aim of obtaining a new hydrazone series with a wide use in medicinal applications, herein we report the synthesis of a new series of hydrazones together with the evaluation of their antioxidant activities.

## Results and Discussion

### Chemistry

The new analogues of hydrazone derivatives were synthesized, characterized, and investigated for their potential antioxidant activities by free radical scavenging, reducing power, and metal chelating test models. The flexible alkyl chains of the hydrazone compounds were changed systematically.

In here, we report the reaction of 4-alkoxy-2-hydroxybenzaldehyde (**1a-f**) and *p*-nitro-phenylhydrazine (**2**) with a *p*-toluenesulfonic acid catalyst in toluene to form the hydrazone derivatives **3a-f** (Scheme 1). In this reaction, the starting materials (**1a-f** and **2**) were used in a 1:1 molar equivalent and purified by crystallization from acetone/ethanol.

By IR, ^1^H-,^13^C-NMR spectra, and MS data, the structures of the hydrazone derivatives were characterized.

The IR spectrum of these compounds showed absorption bands at 1598 cm^-1^ (–C=N-stretching). Thus, the ^1^H-NMR spectra showed a singlet at δ 7.90–7.91 for the –HC=N-protons and broad singlets at δ 7.78–7.86 for the NH groups. The presence of –C=N-NH- in the molecule was also shown by its ^13^C-NMR spectrum which showed peaks at δ 145.25–145.43 (C=N).

**Sch. 1.**
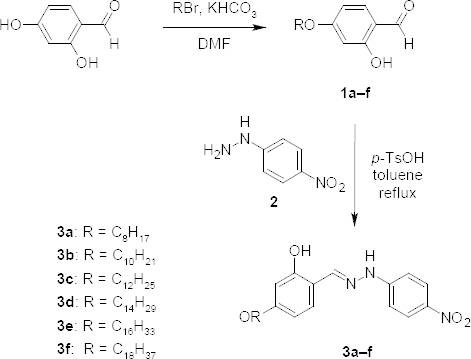
Synthetic route of hydrazone compounds (**3a–f**)

As a result, the spectral data of the synthesized compounds were found to be consistent with the expected structure (see “Experimental” section).

### Antioxidant Activities

All the new hydrazone compounds **3a–f** were evaluated for their antioxidant activity. The free radical-scavenging activity of the synthesized compounds was determined by 1,1-di-phenyl-2-picrylhydrazyl (DPPH) and 2,2’-azino-bis(3-ethylbenzothiazoline-6-sulfonic acid) (ABTS). Reduction of Fe^+3^ to Fe^+2^ was tested by measuring absorbance. The chelating activity of the new series of the hydrazone-derived salicylaldehyde was evaluated against Fe^2+^.

The hydrazone compounds have a certain proportion of antioxidant activity as a result of the presence of the -NH-N = group. A lot of research on antioxidant activity studies of Schiff bases, hydrazone compounds, and semicarbazones is present in the literature [13–17], but systematic studies on the alternating alkyl chains of phenylhydrazone compounds is firstly reported by our group in this paper.

### Free Radical-Scavenging Activity

DPPH^•^ Scavenging Activity

The 1,1-diphenyl-2-picryhyrazyl radical (DPPH^•^) is a stable free radical, which surveys the antioxidant potential of pure compounds by an easy colorimetric method based on the guideline that accepting a hydrogen atom from the antioxidant scavenger compound DPPH^•^ turns to DPPH_2_, and the change in colour from purple to yellow is screened spectrophotometrically at 517 nm [18, 19]. Higher free radical scavenging activity is designated by a lower absorbance of a DPPH^•+^ sample mixture measured at the concerned wavelength.

**Fig. 1.**
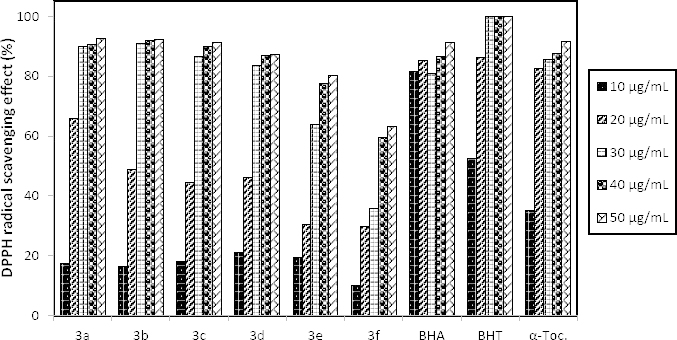
The DPPH^•^ scavenging activity percentages at various concentrations of the synthesized hydrazone compounds (**3a–f**)

Figure 1 depicts the percentage of antioxidant activity at various concentrations of the synthesized hydrazone compounds. All the compounds (**3a–f)** showed high free radical-scavenging activity by inhibiting the DPPH radical in a concentration-dependent manner. Compound **3a,** by having the shortest alkoxy chain, exhibited the best antioxidant activity of 92.42% which was higher than the reference antioxidants, butylated hydroxyanisole (BHA) and α-tocopherol at the concentration of 50 µg/mL. The other reference was butylated hydroxytoluene (BHT). Interestingly, it was determined that the antioxidant activity of the compounds decreased as the alkoxy chains lengthened. As a result of it, compound **3f** showed the lowest antioxidant capacity of 63.31% due to its longest alkoxy chain.

ABTS^•+^ Scavenging Assay

**Fig. 2.**
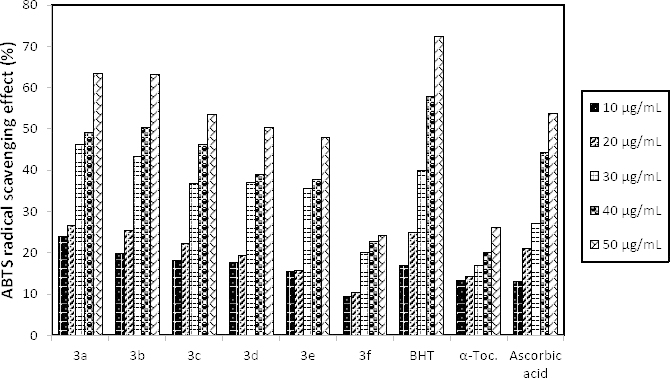
The ABTS^•+^ scavenging activity percentages at various concentration of the synthesized hydrazone compounds (**3a–f**)

The ABTS^•+^ scavenging activity was carried out according to Arnao’s method. It is a decolourization method incapsulating preformed ABTS^•+^ by combining 2.6 mM potassium persulfate as an oxidant with ABTS at the concentration of 7.8 x 10^-3^ mM in distilled water. This results in a dark blue or green stable radical solution after 12–16 h of incubation in the dark. According to the method, methanol was used to dilute the radical solution to give an absorbance at 734 nm of 1.1±0.02 in a cuvette to form the test reagent. Absorption decreases as the antioxidant capability of the compounds increases, just like the other decolourization methods. Figure 2 represents the ABTS^•+^ scavenging activity of the synthesized compounds.

The results of hydrazone compounds **3a–f** showed antioxidant activities providing their capacity to scavenge the ABTS^•+^ radical cation at a range of 10–50 µg/mL. Although the DPPH and ABTS radical scavenging is based on the same principle, the data obtained from ABTS are lower than those obtained from the DPPH activity. Even so, the results of the ABTS assay are in a concentration–dependent manner just like in the DPPH scavenging activity. Compounds **3a** and **3b** exhibited antioxidant capacities of 63.49% and 63.13% which are higher than the reference antioxidants, ascorbic acid and α-tocopherol (53.82% and 26.09%, respectively) at the concentration of 50 µg/mL. Results of the compounds **3c**, **3d**, and **3e** (53.5%, 50.23%, and 47.92%, respectively) are very similiar while comparing to that of ascorbic acid (53.82%) at the same concentration. At the concentration of 50 µg/mL, compound **3f** has the lowest radical scavenging activity with an antioxidant capacity of 24.28%, but even so, it is approximate to the value of α-tocopherol (26.09%).

### Ferric Reducing Antioxidant Power (FRAP)

Oyaizu’s method was chosen to measure the reducing power of the new compounds. According to the reduction of Fe^+3^ to Fe^+2^, the ferric reducing antioxidant power of the synthesized hydrazone compounds was determined by the absorbance-measuring of Perl’s Prussian blue complex.

**Fig. 3.**
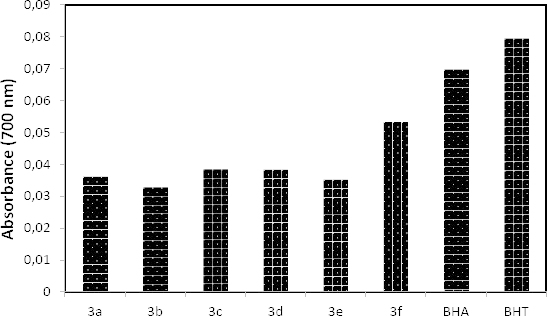
The reducing power capacity when compared to that of the standard BHT and BHA at the concentration 10 µg/mL of hydrazone compounds (**3a–f**)

Figure 3 shows the reducing power of compounds **3a-f**. The synthesized compounds showed moderate reducing power capacity when compared to that of the standard BHT and BHA at the concentration of 10 µg/mL. The reducing power assay revealed that the assayed compounds were able to reduce the ferric ions (Fe^+3^) to ferrous ions (Fe^+2^) at this concentration.

### Ferrous Ion (Fe^+2^) Chelating Activity

The metal chelating activity of the new *p*-nitrophenylhydrazone derivatives **3a–f** on Fe^2+ ^was studied according to Decker and Welch’s method. Prior to the assay, a coloured complex is formed by ferrozine binding to Fe^2+^. Through the Fenton reaction, the countenance of lipid peroxidation generating hydroxyl radicals by the Fe ion is executed and accelerates lipid peroxidation into peroxyl and alkoxyl radicals [20]. Free radical damage could be reduced by chelating agents due to their ability of stabilizing transition metals by causing the inhibition of radical generation [21].

The chelating activity of a new series of hydrazone-derived salicylaldehyde was evaluated against Fe^2+^ for an estimate of the potential antioxidant activity.

**Fig. 4.**
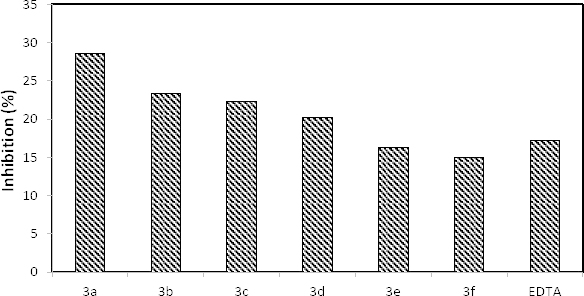
Fe^2+^ chelating activities of the new series of hydrazones (**3a–f**)

The metal chelating activities of compounds **3a–f** are presented in Figure 4. The chelating ability order of the test samples from maximum to minimum was found to be 28.59%, 23.3%, 22.29%, 20.15%, 16.33%, and 14.96%, respectively, at a concentration of 20 µg/mL. It is seen that the results of compounds **3a–d** are better in metal chelating activity than that of EDTA (17.14%) and **3e–f** are moderate. From that point of view, after toxicological test systems, compounds **3a–f** can be used as standard materials for chelating agents like EDTA.

### Antioxidant Activity by the Ferric Thiocyanate Method

Total antioxidant activity determination was done by measuring the absorbance at 500 nm according to the thiocyanate method. The ability of antioxidants to scavenge peroxyl radicals through hydrogen donation during polyunsaturated fatty acid (PUFA) oxidation was determined by the ferric thiocyanate method. In here, the thiocyanate group reacts with Fe^+3^ and the red color of the complex was observed with the oxidation of Fe^+2^ by peroxides, and the change in absorbance was measured every 24 h until the reaction was finished.

**Fig. 5.**
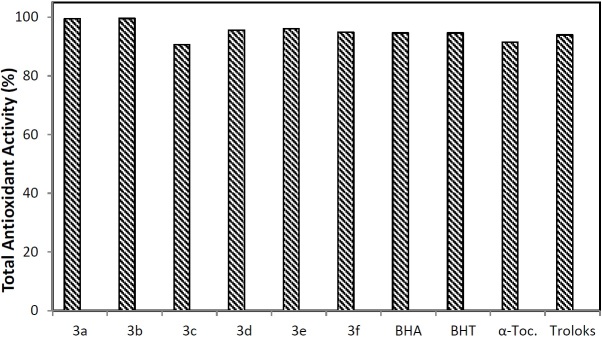
Total antioxidant activity percentages of the new hydrazone compounds (**3a–f**)

The effects of the compounds in preventing the peroxidation of linoleic acid are shown in Figure 5. The oxidation of linoleic acid was reduced in the presence of the newly synthesized hydrazone derivatives. The results clearly indicate that all the compounds exhibited significant antioxidant activity.

## Experimental

### Materials and Methods

The standard chemicals were supplied by Fluka, Sigma-Aldrich GmbH, Merck Chemical Company, used without further purification. All solvents were also supplied by the Merck Chemical Company and purified by standard purification methods.

The melting points were determined by the digital Gallenkamp Melting Point Apparatus in open capillaries. The molecular structures of the new hydrazone compounds were confirmed by various spectroscopic data as IR (Perkin Elmer FT-IR Spectrophotometer in 4000–400 cm^-1^ using KBr pellets), ^1^H-NMR, ^13^C-NMR (Varian Unity 400 Spectrometer in CDCI3 and using TMS as the internal standard), and MS (Varian MAT 711). The absorbance values in the antioxidant activity studies were measured by the Lambda 25 Spectrophotometer (Perkin Elmer).

### Synthesis of the Hydrazone Compounds 3a-f

The hydrazone compounds **3a-f** were obtained and characterized using the same condensation procedure as reported earlier by our group [22, 23]. The new compounds (**3a-f**) were prepared by the *p*-toluensulfonic acid (40 mg)-catalyzed condensation of 4-alkoxy-2-hydroxybenzaldehyde **1a-f** (2.5 mmol) with 4-nitrophenylhydrazine **2** (3 mmol) in toluene (40 ml). The crystallization from acetone/ethanol was performed to purify the compounds.

#### 2-{[2-(4-Nitrophenyl)hydrazinylidene]methyl}-5-(octyloxy)phenol (3a)

Orange crystals. Yield: 0.72 g, 75%, m.p. 143.0°C; IR (KBr) cm^-1^ 1598 (C=N, azomethine stretch). ^1^H-NMR (400 MHz, CDCl3, TMS) δ: 10.54 (s; OH), 8.17 (d, *J =* 8.1 Hz; 2 Ar-H), 7.90 (s; HC=N), 7.80 (broad s; NH), 7.06 (d; *J =* 8.5 Hz; 1 Ar-H), 6.93 (d, *J =* 9.1 Hz; 2 Ar-H), 6.52 (d, *J =* 2.3 Hz; 1 Ar-H), 6.48 (dd, *J =* 2.3 Hz, *J =* 8.5 Hz; 1 Ar-H), 3.96 (t; *J =* 6.6 Hz, -OCH2 group), 2.51–1.62 (m; 6 CH_2_), 0.61 (t; *J =* 6.6 Hz, –CH3 group). ^13^C-NMR (100 MHz, CDCl3, TMS) δ: 162.17, 159.28, 148.43, 140.60, 131.31, 126.31, 111.29, 107.68, 102.04 (Ar–C), 145.39 (HC=N), 68.33 (OCH_2_), 34.11–22.90 (CH_2_), 14.16 (CH_3_). C_21_H_27_N_3_O_4_ (385.457). MS *m/z* (%) = 385 [M^+^, 100 %].

#### 5-(Decyloxy)-2-{[2-(4-nitrophenyl)hydrazinylidene]methyl}phenol (3b)

Orange crystals. Yield: 0.79 g, 78.6%, m.p. 141.6°C; IR (KBr) cm^-1^ 1598 (C=N, azomethine stretch). ^1^H-NMR (400 MHz, CDCl3, TMS) δ: 10.53 (s; OH), 8.18 (d, *J =* 8.1 Hz; 2 Ar-H), 7.90 (s; HC=N), 7.79 (broad s; NH), 7.06 (d; *J =* 8.5 Hz; 1 Ar-H), 6.93 (d, *J =* 9.1 Hz; 2 Ar-H), 6.52 (d, *J =* 2.3 Hz; 1 Ar-H), 6.48 (dd, *J =* 2.3 Hz, *J =* 8.5 Hz; 1 Ar-H), 3.96 (t; *J =* 6.6 Hz, -OCH2 group), 2.51–1.60 (m; 8 CH_2_), 0.60 (t; *J =* 6.6 Hz, –CH3 group). ^13^C-NMR (100 MHz, CDCl3, TMS) δ: 162.08, 159.20, 148.33, 140.52, 131.27, 126.29, 111.25, 107.65, 101.97 (Ar–C), 145.29 (HC=N), 68.31 (OCH_2_), 34.15–23.00 (CH_2_), 14.14 (CH_3_). C_23_H_31_N_3_O_4_ (413.510). MS *m/z* (%) = 413 [M^+^, 100 %].

#### 5-(Dodecyloxy)-2-{[2-(4-nitrophenyl)hydrazinylidene]methyl}phenol (3c)

Orange crystals. Yield: 0.80 g, 72.9%, m.p. 130.6°C; IR (KBr) cm^-1^ 1598 (C=N, azomethine stretch). ^1^H-NMR (400 MHz, CDCl3, TMS) δ: 10.52 (s; OH), 8.18 (d, *J =* 8.1 Hz; 2 Ar-H), 7.90 (s; HC=N), 7.83 (broad s; NH), 7.06 (d; *J =* 8.5 Hz; 1 Ar-H), 6.93 (d, *J =* 9.1 Hz; 2 Ar-H), 6.52 (d, *J =* 2.3 Hz; 1 Ar-H), 6.48 (dd, *J =* 2.3 Hz, *J =* 8.5 Hz; 1 Ar-H), 3.97 (t; *J =* 6.6 Hz, -OCH2 group), 2.52–1.61 (m; 10 CH_2_), 0.61 (t; *J =* 6.6 Hz, –CH3 group). ^13^C-NMR (100 MHz, CDCl3, TMS) δ: 162.07, 159.18, 148.27, 140.58, 131.22, 126.24, 111.27, 107.67, 102.01 (Ar–C), 145.25 (HC=N), 68.35 (OCH_2_), 34.14–23.01 (CH_2_), 14.14 (CH_3_). C_25_H_35_N_3_O_4_ (441.564). MS: *m/z* (%) = 441 [M^+^, 100 %].

#### 2-{[2-(4-Nitrophenyl)hydrazinylidene]methyl}-5-(tetradecyloxy)phenol (3d)

Orange crystals. Yield: 0.91 g, 77.2%, m.p. 128.5°C; IR (KBr) cm^-1^ 1598 (C=N, azo-methine stretch). ^1^H-NMR (400 MHz, CDCl_3_, TMS) **δ**: 10.53 (s; OH), 8.18 (d, *J =* 8.1 Hz; 2 Ar-H), 7.91 (s; HC=N), 7.78 (broad s; NH), 7.06 (d; *J =* 8.1 Hz; 1 Ar-H), 6.93 (d, *J =* 8.9 Hz; 2 Ar-H), 6.52 (d, *J =* 2.3 Hz; 1 Ar-H), 6.48 (dd, *J =* 2.3 Hz, *J =* 8.5 Hz; 1 Ar-H), 3.96 (t; *J =* 6.6 Hz, -OCH2 group), 2.51–1.61 (m; 12 CH_2_), 0.60 (t; *J =* 6.6 Hz, –CH3 group). ^13^C-NMR (100 MHz, CDCl3, TMS) **δ**: 162.12, 159.25, 148.38, 140.52, 131.31, 126.33, 111.26, 107.66, 101.96 (Ar–C), 145.33 (HC=N), 68.29 (OCH_2_), 34.15–23.04 (CH_2_), 14.13 (CH_3_). C_27_H_39_N_3_O_4_ (469.617). MS: *m/z* (%) = 469 [M^+^, 100 %].

#### 5-(Hexadecyloxy)-2-{[2-(4-nitrophenyl)hydrazinylidene]methyl}phenol (3e)

Orange crystals. Yield: 1.03 g, 82.8%, m.p. 127.2°C; IR (KBr) cm^-1^ 1598 (C=N, azo-methine stretch). ^1^H-NMR (400 MHz, CDCl3, TMS) **δ**: 10.54 (s; OH), 8.17 (d, *J =* 8.1 Hz; 2 Ar-H), 7.91 (s; HC=N), 7.86 (broad s; NH), 7.06 (d; *J =* 8.6 Hz; 1 Ar-H), 6.93 (d, *J =* 9.1 Hz; 2 Ar-H), 6.52 (d, *J =* 2.3 Hz; 1 Ar-H), 6.47 (dd, *J =* 2.3 Hz, *J =* 8.5 Hz; 1 Ar-H), 3.96 (t; *J =* 6.6 Hz, -OCH2 group), 2.53–1.59 (m; 14 CH_2_), 0.62 (t; *J =* 6.6 Hz, –CH3 group). ^13^C-NMR (100 MHz, CDCl3, TMS) **δ**: 162.03, 159.16, 148.36, 140.47, 131.22, 126.23, 111.24, 107.63, 102.01 (Ar–C), 145.30 (HC=N), 68.35 (OCH_2_), 34.15–23.03 (CH_2_), 14.13 (CH_3_). C_29_H_43_N_3_O_4_ (497.670) MS: *m/z* (%) = 497 [M^+^, 100 %].

#### 2-{[2-(4-Nitrophenyl)hydrazinylidene]methyl}-5-(octadecyloxy)phenol (3f)

Orange crystals. Yield: 1.10 g, 83.9%, m.p. 125.1°C; IR (KBr) cm^-1^ 1598 (C=N, azo-methine stretch). ^1^H-NMR (400 MHz, CDCl3, TMS) **δ**: 10.54 (s; OH), 8.17 (d, *J =* 8.1 Hz; 2 Ar-H), 7.90 (s; HC=N), 7.80 (broad s; NH), 7.06 (d; *J =* 8.5 Hz; 1 Ar-H), 6.93 (d, *J =* 9.1 Hz; 2 Ar-H), 6.52 (d, *J =* 2.3 Hz; 1 Ar-H), 6.48 (dd, *J =* 2.3 Hz, *J =* 8.5 Hz; 1 Ar-H), 3.96 (t; *J =* 6.6 Hz, -OCH2 group), 2.52–1.60 (m; 16 CH_2_), 0.62 (t; *J =* 6.6 Hz, –CH3 group). ^13^C-NMR (100 MHz, CDCl3, TMS) **δ**: 162.17, 159.28, 145.39, 140.60, 131.31, 126.31, 111.29, 110.89, 107.68, 102.04 (Ar–C), 145.43 (HC=N), 68.33 (OCH_2_), 34.15–23.03 (CH_2_), 14.14 (CH_3_). C_31_H_47_N_3_O_4_ (525.723) MS*: m/z (%) =* 525 [M^+^, 100 %].

### In Vitro Antioxidant Activity Assay

#### DPPH^•^ Scavenging Assay

The effect of the new *p*-nitrophenylhydrazone derivatives **3a-f** on DPPH^•^ was detected by using the procedure described by S´anchez-Moreno et al. [24].

BHA, BHT, and a-tocopherol standards were used to compare the scavenging effect of the compounds and the scavenging activity of all the tested molecules calculated by the formula (A_o_ - A_1_) \ A_o_ X 100 where A_o_ is the absorbance of the control reaction and A_1_ is the absorbance in the presence of the samples.

#### ABTS^•+^ Scavenging Assay

The ABTS^•+^ scavenging activity was carried out according to Arnao’s method [25]. Once radical electrons are trapped by antioxidants in the samples, the color of the test solution disappears gradually and the absorbance at 734 nm is reduced. BHT, a-tocopherol, and ascorbic acid served as standards to compare with the activities of the compounds.

#### Reducing Power

Oyaizu‘s method [26] was chosen to measure the reducing power of the new compounds. Higher absorbance indicates a higher reducing power of the compounds by comparing them with specific standards such as BHA, BHT, and α-tocopherol.

#### Metal Chelating Activity

The metal chelating activity of the new *p*-nitrophenylhydrazone derivatives **3a–f** on Fe^2+ ^was studied according to Decker and Welch’s method [27]. In this method, a lower absorbance shows a stronger Fe^2+^ chelating ability by comparing EDTA as a positive control.

#### Antioxidant Activity by the Ferric Thiocyanate Method

Total antioxidant activity determination was done by measuring the absorbance at 500 nm according to the thiocyanate method [28]. The absorbance of the control solution of this process was measured every 24 h until it reached a maximum value.

## Conclusion

In the present study, a new series of phenylhydrazone derivatives have been successfully synthesized and characterized by using various spectroscopic methods. The synthesized compounds were investigated by the scavenging effect on the DPPH^•^ and ABTS^•+^ radicals, reducing power, metal chelating activity, and total antioxidant activity. All the hydrazone compounds showed good antioxidant activity because of the combination of different alkyl chains with hydrazone functional groups in the molecule structure. This combination can stabilize the free radicals and among the hydrazone compounds, **3a** had the highest antioxidant activity. The synthesized hydrazones in this study were moderate radical scavengers, having similar activity to that of BHA and BHT. In addition to these, the hydrazone compounds were more successful in chelating with ferrous ions. These compounds also showed important antioxidant activities in blocking the heat-induced oxidation of linoleic acid.
